# Overexpression of Recombinant Human Teriparatide, rhPTH (1–34) in *Escherichia coli* : An Innovative Gene Fusion Approach

**Published:** 2017

**Authors:** Nahid Bakhtiari, Zahra Amini Bayat, Sepideh Sagharidouz, Mohsen Vaez

**Affiliations:** Department of Biotechnology, Iranian Research Organization for Science and Technology (IROST), Tehran, Iran

**Keywords:** Cloning, *Escherichia coli*, Expression, Recombinant, Teriparatide

## Abstract

**Background:**

Parathyroid hormone is an 84-amino acid peptide secreted by the parathyroid glands. Its physiological role is maintenance of normal serum calcium level and bone remodeling. Biological activity of this hormone is related to N-terminal 1–34 amino acids. The recombinant form of hormone (1–34) has been approved for treatment of osteoporosis from 2002. In this study, a novel fusion partner has been developed for preparation of high yield recombinant 1–34 amino acids of hPTH.

**Methods:**

Novel nucleotide cassette designed encoding a chimeric fusion protein comprising of a fusion partner consisting of a His-tag in N-terminal, 53 amino acids belong to *Escherichia coli (E. coli)* β-galactosidase (LacZ) gene, a linker sequence for increasing of expression and protection of target peptide structure from fusion tag effect, an Enteropeptidase cleavage site, rhPTH (1–34) gene fragment. Optimized fusion gene was synthesized and ligated into pET-28a vector under control of T7 promoter, and then transformed in *E. coli* (DHα) cells. Positive clones containing this gene were double digested with NcoI and-BamHI and also approved by sequencing. Gene overexpression was observed in SDS-PAGE after induction with 0.2 *mM* IPTG. Confirmation of gene expression was performed by western blotting using anti-His-tag antibody conjugated with peroxidase.

**Results:**

By this fusion gene design approach, we achieved a high level expression of the rhPTH, where it represented at least 43.7% of the total protein as determined by SDS-PAGE and confirmed by western blotting.

**Conclusion:**

In addition to high level expression of the designed gene in this work, specific amino acid sequence of bacterial β-galactosidase was selected as major part of carrier tag for protection of this hormone as important step of recombinant rhPTH with relevant isoelectronic point (pI). This innovation resulted in recombinant production of hPTH very well and the gene construct could be applied as a pattern for similar recombinant peptides where recombinant protein degradation is a critical issue.

## Introduction

Maintenance of calcium/phosphate equilibrium and bone resorption regulation in human body are very significant processes that affected by different parameters. Parathyroid hormone consisted of 84 amino acids and secreted by parathyroid glands which is among the main mechanisms of the body that involve in regulation of such a complex process 1. Bone regeneration process takes 3 to 6 months and involves the coupling of bone resorption and formation pathways. If for any reason this process fails, gradually the person will suffer from osteoporosis 1,2. In this condition, bone mass decreases and the patient is prone to fracture. Human recombinant Teriparatide (rhPTH) passed FDA approval to be applied for treatment of this disease from 2002 3. Teriparatide, is a peptide drug contains 1–34 N- terminal amino acids (the minimum length of active parathormone) of full length 84 amino acid parathyroid hormone 4. The recombinant 34 amino acid hPTH which produced in *Escherichia coli (E. coli)* has an unstable structure and degradation immediate after expression. Different researchers have solved this problem by construction of different fusion proteins 5. Fusion elements generally used contain GST, phosphoribulokinase, TRX, hGH, cro- β-galactosidase and β-galactosidase 6–11. Different parts of β-galactosidase gene have been used as fusion collaborator in different recombinant proteins. 20 to 500 *mg/l* rhPTH were obtained when different lengths of this fusion partner have been applied 11. In this study, a novel fusion gene was designed for gene overexpression and purification with feasible and economical methods. For this purpose, amino acid sequence 236 to 288 of *E. coli* K12 β-galactosidase gene included beta sheets structure, was used as carrier fusion tag to protect recombinant teriparatide from degradation. A polyhistidine leader peptide was included in 5′ end of fusion gene for primary isolation of overexpressed gene from bacterial proteins. In addition, an enteropeptidase recognition site was applied for the cutting of 34 amino acids teriparatide from its fusion partner and secondary purification with cation exchange chromatography. High level expression of the designed fusion gene, at least 43.7% of total protein of *E. coli*, was obtained in this research project.

## Materials and Methods

### Strains, media and culture conditions

*E. coli* DH5α strain (F- 80dlacZ M15 (lacZYA-argF) U169 recA1 endA1hsdR17 (rk−, mk+) phoAsup E44 -thi-1 gyrA96 relA1) was used as cloning host and plasmid amplification. *E. coli* BL21 (DE3) (F- ompT gal dcm lon hsdSB (rB- mB-) λ (DE3 [lacI lacUV5-T7 gene 1 ind1 sam7 nin5])) (Studier, 1986) was employed for expression of desired fusion protein. Luria-Bertani (LB) (Liofilchem) with 50 *μg/ml* Kanamycin (Duchefa Biochemie) was used for bacteria growth at 37°*C* and 200 *rpm*.

### Design and construction of expression vector harboring rhPTH (1–34) fusion gene

**Figure 1.**
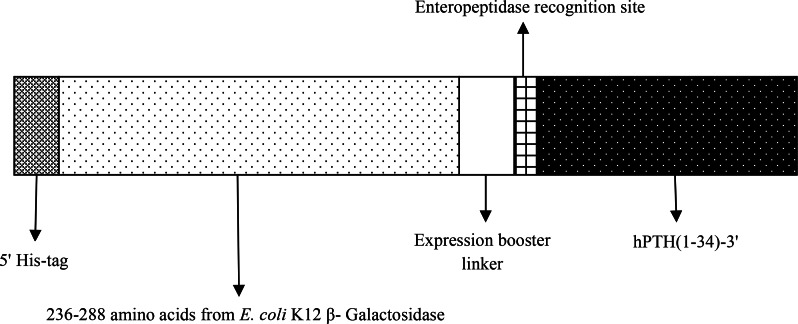
Schematic of designed fusion hPTH (1–34).

A fusion ORF of rhPTH consisting 359 *bp* was designed including NcoI-BamHI as restricted frame containing his tag, 159 *bp* sequence of bacterial β-galactosidase along with expression booster linkers, enteropeptidase cleavage site and the sequences encoded for rhPTH (1–34). The designed gene was synthesized by Generay Biotech Co. (Shanghai, China) and digested with NcoI and BamHI (Thermo Scientific) in 37°*C* for 1 *hr* and purified with spine column DNA gel extraction kit (Bio Basic) after gel electrophoresis. pET-28a plasmid was double digested with same restriction enzymes and gene insertion and ligation were done using fast DNA ligation kit (Bio Basic). Competent *E. coli* (DH5α) cells were transformed with recombinant cloning vector containing the fusion ORF. Recombinant plasmids recognized with double digestion and DNA sequencing. Schematic figure of fusion hPTH (1–34) has been shown in figure 1.

### Expression of fusion rhPTH (1–34) protein in E. coli BL21 (DE3)

Transformed *E. coli* BL21 (DE3) with recombinant vector were grown overnight in LB medium containing 50 *μg/ml* Kanamycin at 37°*C* with shaking rate of 200 *rpm* until log phase of grown with addition of 0/2 *mM* IPTG (Fermentas) to induce the production of rhPTH at 28°*C* and the outcomes were monitored in 16 *hr*. The Recombinant protein expression was evaluated in 12.5% SDS-PAGE gel. Voltage used for stacking gel was 80 *V* and resolving gel was run at 120 *V*. Coomassie blue (Merck) staining method was applied to observe protein bands.

### Western blotting assay of fusion hPTH (1–34)

Expressed proteins were transferred to 0/2 *μm* pore size nitrocellulose membrane (Sigma-aldrich) at 100 *mA* for 3 *hr* and detected with Horseradish Peroxidase conjugated anti His-tag antibody (Sigma-aldrich) and visualized by DAB (BIO BASIC) staining method.

### Protein yield and rhPTH fusion protein expression level

Concentration of expressed fusion protein by bacteria was calculated with AlphaEase FC software.

## Results

### Design and construction of expression vector harboring rhPTH (1–34) fusion gene

**Figure 2.**
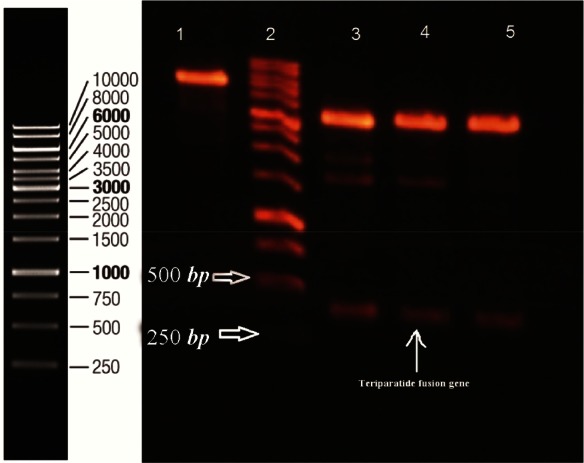
Double digestion of recombinant plasmid with Nco1 and BamH1. Lane 1 undigested pET 28a. Lane 2 DNA ladder (Thermo scientific). 3, 4, 5 double digested pET 28a. Designed fusion rhPTH (1–34) is observed between 500 and 250 *bp* ladder bonds.

Specific β-sheet structure of bacterial β-galactosidase including amino acid 236–288 was selected as major part of carrier tag for protection of rhPTH (1–34) against degradation. The pI of 5.18 of designed fusion partner compared to 8.29 of rhPTH (1–34) was in accordance with our goal for high yield separation of recombinant teriparatide hormone from its fusion sections using ion exchange chromatography method. The synthesized rhPTH (1–34) fusion gene was digested with NcoI and BamHI and ligated with pET-28a digested with same restriction enzymes. Recombinant plasmids were recognized with double digestion (Figure 2). Verification of inserted gene in pET-28a was performed by sequencing for rhPTH (1–34) fusion gene.

### Expression of fusion rhPTH (1–34) protein in E. coli BL21 (DE3)

**Figure 3.**
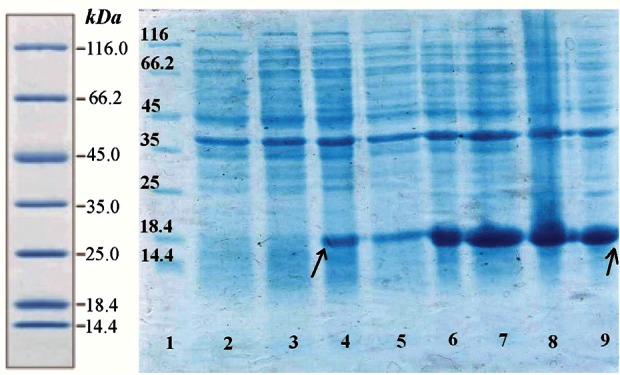
Analysis of expression pattern of rhPTH (1–34) fusion protein with SDS-PAGE. Lane 1 protein ladder (Thermo scientific). Lane 2 Un-induced BL21 (DE3). Lane 4 one hour after induction with IPTG (0.2 *mM*). Lane 6 four hours after induction. Lane 8 sixteen hours after induction. Over expressed fusion rhPTH (1–34) bonds have specified with rows.

**Figure 4.**
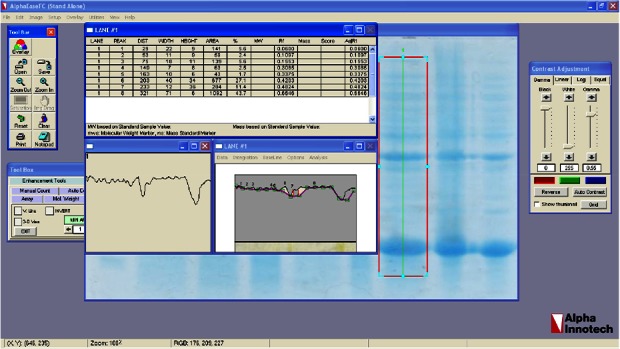
calculation of expression yield of Teriparatide fusion gene with AlphaEase FC software.

Suitable expression of desired protein is the first step in recombinant protein production. Expressed fusion protein, rhPTH, was observed in location of 18.4 *kDa* band of marker in SDS-PAGE gel (Figure 3), and the calculations of pI/Mw by expasy showed the molecular weight of 12.8 *kDa*. Such differences already been reported in other studies and explained due to compact and negatively charged of fusion rhPTH protein. This structure prevents sufficient SDS binding leading to lower charge to mass ratio and mobility in SDS-PAGE gel 12. Fusion protein was also shown to be stably expressed in *E. coli* BL21 (DE3) for up to 16 *hr* after induction at 28°*C*. The expression quantity of rhPTH (1–34) was at least 43.7% of total protein of *E. coli* BL21 (DE3) calculated with AlphaEase FC software (Figure 4)

### Western blotting assay of hPTH fusion protein

**Figure 5.**
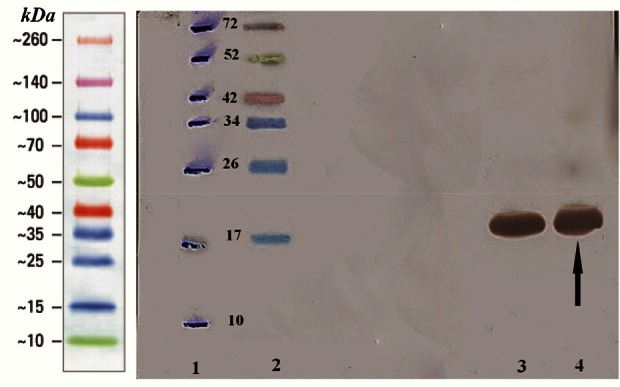
Analysis of over expressed recombinant fusion rhPTH (1–34) with western blotting. Lane 1 marked off protein ladder bonds with pen. Lane 2 Multicolor protein ladder (Thermo scientific). Lane 3, 4 over expressed recombinant fusion rhPTH (1–34) revealed with monoclonal anti-polyhistidine peroxidase conjugate.

The fusion rhPTH protein bands on SDS-PAGE gel were confirmed with western blotting using nitrocellulose membrane and anti his-tag antibody (Figure 5). The molecular weight of fusion protein was reconfirmed at 18 *kDa* band.

## Discussion

Different fusion genes constructs have been already designed for rhPTH production in *E. coli*. In these studies, fusion partners including hGH, GST, phosphoribulokinase, cro- β-galactosidase, TRX and β-galactosidase have been used 6–11. For fusion partner cleavage chemical substances such as diluted HCl and cyanogen bromide as well as different proteases as TEV protase, Kex2, Thrombin, Prolyl Endopeptidase (PE) and enterokinase are applied. Various methods have been designed for rhPTH extraction and purification from *E. coli*. However, there have been several difficulties in these procedures such as low level of recombinant protein expression, environmental contamination with cyanogen bromide and using chemical substances and application of enzymes for rhPTH cleavage from fusion partner such as Kex2 or using of two protease are not economical 6,9,13–17. Purification complexity is another concern in recombinant rhPTH production. In this study, high level expression of recombinant protein around 50% out of whole protein shows efficient production.

In addition, an Enteropeptidase recognition sequence has been embedded at the N-terminal of rhPTH for cleavage of fusion protein after the initial purification step. This enzyme is a cost effective even for scaling up of production process. An N-terminal His-tag has been used for initial isolation of recombinant fusion protein bacterial cell lysates. As a conclusion, the fusion tag approach with different pI for rhPTH (1–34) could provide a suitable method for an easy and efficient final purification of recombinant active rhPTH (1–34).

## Conclusion

In this study, fusion gene design with the aim of obtaining high level of rhPTH expression and purity in *E. coli* was carried. For this purpose, affinity and carrier tags, special linkers and protease cutting site were designed inside the nucleotide sequence of gene construct. Finally, an innovative gene fusion approach with high level of gene expression presented here could be applied for production of similar peptides.
